# Roles and Outcomes of Advanced Practice Nurses in Thailand: A Systematic Review

**DOI:** 10.3390/healthcare14152300

**Published:** 2026-07-29

**Authors:** Phatcharaphon Whaikid, Noppawan Piaseu

**Affiliations:** 1 Faculty of Nursing, Huachiew Chalermprakiet University, Samut Prakan 10540, Thailand; phatcharaporn.wha@lhcu.ac.th; 2 Ramathibodi School of Nursing, Faculty of Medicine Ramathibodi Hospital, Mahidol University, Bangkok 10400, Thailand; 3 Center for Health Promotion and Well-Being, Faculty of Medicine Ramathibodi Hospital, Mahidol University, Bangkok 10400, Thailand; 4 JBI Evidence-Based Healthcare Ramathibodi School of Nursing Center, University of Adelaide, North Adelaide, SA 5006, Australia

**Keywords:** advanced practice nursing, healthcare outcomes, holistic nursing, systematic review, Thailand

## Abstract

**Background and Objectives:** Advanced practice nurses (APNs) play an increasingly important role in improving healthcare quality and outcomes. This systematic review synthesized evidence on APN roles and healthcare outcomes in Thailand. **Methods:** The review protocol was prospectively registered in PROSPERO (CRD420261331793). Four databases (PubMed, CINAHL, Scopus, and Thai Journals Online) were searched from inception to January 2026. Two independent reviewers performed study selection, data extraction, and methodological quality assessment using the Mixed Methods Appraisal Tool (MMAT, 2018) for mixed-methods studies and the Joanna Briggs Institute (JBI) critical appraisal checklists for quasi-experimental, analytical cross-sectional, and qualitative studies. A narrative synthesis was undertaken because of methodological heterogeneity. **Results:** Twenty studies were included, comprising eight quasi-experimental studies, six cross-sectional studies, four mixed-methods studies, one retrospective study, and one qualitative descriptive study. APNs performed diverse roles, including direct clinical care, care coordination, consultation, patient education, mentoring, and implementation of evidence-based practice. APN-led models of care were associated with favorable clinical outcomes, enhanced self-care and quality of life, increased patient and caregiver satisfaction, and reduced hospital length of stay and healthcare costs. **Conclusions:** The available evidence suggests that APN-led models of care may be associated with favorable patient, family, and health system outcomes in Thailand. However, these findings should be interpreted cautiously because the evidence was derived predominantly from non-randomized and observational studies, and the independent effects of APN practice could not always be isolated.

## 1. Introduction

Global health systems are increasingly challenged by population aging, multimorbidity, chronic disease burden [1], and persistent workforce shortages [2], highlighting the urgent need for advanced nursing roles and more flexible models of care [3]. Advanced practice nursing (APN) has emerged globally as a key strategy to address growing healthcare demands, workforce shortages, and the increasing complexity of patient care [4]. APNs are registered nurses who have acquired expert knowledge, complex decision-making skills, and advanced clinical competencies for expanded roles within healthcare systems [5]. These roles often include direct patient care, care coordination, consultation, leadership, education, and the implementation of evidence-based practice [5].

A growing body of international evidence shows that APNs contribute substantially to high-quality, efficient, and patient-centered care. APN practice has been associated with improved clinical outcomes, enhanced access to services, higher patient satisfaction, and reduced healthcare costs [6]. In addition, APNs can effectively manage chronic diseases, support care transitions, and strengthen interdisciplinary collaboration within healthcare teams [7,8]. Importantly, outcomes of APN-led care are often comparable to physician-led care, while offering advantages in accessibility and patient experience [9].

In Thailand, the advanced practice nursing role has been formally recognized and regulated by the Thailand Nursing and Midwifery Council (TNMC) [10]. Thai APNs are master’s-prepared nurses certified by the TNMC who provide advanced clinical care, consultation, and case management. Certified advanced practice nurses are prepared through postgraduate education and board certification, enabling them to provide specialized clinical services across diverse healthcare settings, including hospitals, community health centers, and specialty clinics. Over the past two decades, APN roles in Thailand have expanded across multiple specialties, including medical–surgical nursing, critical care, chronic disease management, psychiatric nursing, and community health [10].

Despite this progress, evidence regarding the effectiveness, scope of practice, and healthcare outcomes of APNs in Thailand remains dispersed across individual studies and professional reports. This fragmentation limits policymakers, educators, and healthcare leaders’ ability to evaluate the full contribution of APNs and to guide future workforce development. To date, no comprehensive systematic review has synthesized the available evidence on APN roles and outcomes in the Thai context.

Although international systematic reviews have demonstrated the positive contributions of APNs, their findings may not be directly applicable to Thailand because APN regulation, scope of practice, healthcare organization, and workforce integration differ across countries. APN roles and outcomes may therefore vary across healthcare systems. A country-specific synthesis is needed to provide contextually relevant evidence for Thailand and to support evidence-informed policy and practice. Therefore, this systematic review aimed to synthesize evidence on the roles of advanced practice nurses and the healthcare outcomes associated with APN practice in Thailand. Information related to implementation barriers and facilitators was synthesized as contextual evidence to support interpretation of the findings.

A clearer understanding of APN contributions is essential to inform national health policy, optimize workforce planning, strengthen advanced nursing education, and support sustainable health system development in Thailand.

## 2. Materials and Methods

### 2.1. Protocol and Registration

This systematic review was conducted to synthesize the existing evidence on the roles and healthcare outcomes of APNs in Thailand. The review was conducted and reported in accordance with the Preferred Reporting Items for Systematic Reviews and Meta-Analyses (PRISMA) 2020 statement [11]. The review protocol was prospectively registered in PROSPERO (CRD420261331793). JBI SUMARI [12] was used for study screening, data extraction, and critical appraisal of the included studies. Narrative synthesis was conducted independently by the review authors due to the heterogeneity of the included studies.

#### Research Question

The research question for this systematic review is as follows: “What roles have advanced practice nurses performed, and what healthcare outcomes have been reported in association with APN practice in Thailand?” This question was developed using the Population, Concept, and Context (PCC) framework recommended in the JBI Manual for Evidence Synthesis [13].

### 2.2. Eligibility Criteria

In this review, APNs were defined exclusively as nurses formally certified by the TNMC. APN status was verified using the information reported in each included study. Only studies explicitly identifying participants as TNMC-certified APNs were eligible for inclusion. Because different professional titles have occasionally been used to describe APN practice in Thailand, professional titles were cross-checked against the reported qualifications and certification status to confirm consistency with TNMC-recognized APN practice.

The inclusion criteria were studies that (1) were original peer-reviewed research articles, (2) were conducted in Thailand, (3) involved TNMC-certified advanced practice nurses, (4) examined APN roles, activities, or APN-led interventions, and (5) reported at least one patient outcome (e.g., clinical outcomes, quality of life, self-care, patient or caregiver satisfaction), healthcare service outcome (e.g., length of stay, readmission, or healthcare costs), or professional/system outcome (e.g., APN role performance, implementation, or organizational outcomes); and (6) employed qualitative, quantitative (including quasi-experimental, cross-sectional, retrospective, or descriptive designs), or mixed-methods study designs. Grey literature (e.g., theses, conference abstracts, reports, or non-peer-reviewed documents) was excluded.

### 2.3. Search Strategy

A comprehensive literature search was conducted in four electronic databases: PubMed, CINAHL, Scopus, and Thai Journals Online (ThaiJO), to identify relevant studies on advanced practice nursing in Thailand. These databases were selected to capture international nursing literature (PubMed, CINAHL, Scopus) and Thai-language local evidence (ThaiJO). The search included studies published from database inception to 31 January 2026 to capture the full range of evidence on the development and implementation of advanced practice nursing roles in the Thai healthcare system. Studies published in English and Thai were included, with no additional language restrictions applied.

The search strategy combined controlled vocabulary terms and free-text keywords related to APN and Thailand using Boolean operators (AND, OR). The core search terms included: (“advanced practice nurse” OR “advanced practice nursing” OR “clinical nurse specialist” OR “nurse practitioner” OR “advanced practice” OR “APN” OR “CNS”) AND (“clinical outcome” OR “health outcome” OR “patient outcome” OR “effectiveness” OR “quality of life” OR “satisfaction” OR “healthcare utilization” OR “length of stay”) AND (“Thailand” OR “Thai”).

### 2.4. Study Selection

The study selection process was conducted in accordance with the Preferred Reporting Items for Systematic Reviews and Meta-Analyses (PRISMA) 2020 statement [11]. One reviewer independently conducted the literature search across the four electronic databases. All identified records were imported into EndNote version 21 (Clarivate, London, UK), where duplicate records were removed. Two reviewers independently screened the titles, abstracts, and full-text articles using the predefined eligibility criteria. Any disagreements or uncertainties regarding study eligibility were resolved through discussion until consensus was reached.

### 2.5. Data Extraction

Data were extracted from the included studies using a predeveloped Microsoft Excel (Microsoft, Redmond, WA, USA). The data extraction form was piloted on a small number of studies and refined before full data extraction. The extracted data included: (1) author and year of publication; (2) study design; (3) type of APN; (4) population/sample; (5) intervention or role of APN; (6) study duration or follow-up; (7) outcomes measured; and (8) key findings.

Two reviewers independently performed data extraction. Any discrepancies were resolved through discussion and consensus.

### 2.6. Methodological Quality Assessment of Studies

To ensure the reliability and validity of this systematic review, the methodological quality of the included studies was assessed using design-specific critical appraisal tools. The Joanna Briggs Institute (JBI) critical appraisal checklists were used for quasi-experimental, descriptive, cross-sectional, and qualitative studies, while the Mixed Methods Appraisal Tool (MMAT) was used for mixed-methods studies. These tools were used to identify potential sources of bias, evaluate methodological rigor, and determine the overall trustworthiness of the included evidence.

The JBI Checklist for Quasi-Experimental Studies, consisting of 9 items, was used. This checklist evaluates the clarity of the cause-and-effect relationship, similarity of participants in comparison groups, appropriateness of control groups, presence of multiple outcome measurements before and after the intervention, completeness of follow-up, consistency of intervention delivery, reliability of outcome measurements, and appropriateness of statistical analysis [14]. For descriptive and cross-sectional studies, the JBI Checklist for Analytical Cross-Sectional Studies, comprising 8 items, was used. This checklist assesses clearly defined inclusion criteria, a detailed description of participants and settings, valid and reliable measurement of exposures and outcomes, identification and management of confounding factors, and the appropriateness of statistical analysis [15].

For qualitative descriptive studies, the JBI Checklist for Qualitative Research, comprising 10 items, was used. This tool examines congruity between methodology, data collection, analysis, and interpretation; the representation of participants’ voices; researcher reflexivity; ethical considerations; and whether conclusions are supported by the data [16]. For mixed-methods studies, the Mixed Methods Appraisal Tool (MMAT), which includes two screening questions and five methodological criteria for each study category, was used. The MMAT assesses the appropriateness of the mixed-methods design, the quality of the qualitative and quantitative components, the adequacy of integration between methods, the interpretation of combined findings, and the management of inconsistencies between qualitative and quantitative results [17].

Two reviewers independently conducted the quality appraisal of all included studies. Any disagreements were resolved through discussion to reach a consensus. No studies were excluded solely on methodological quality; however, appraisal findings were considered during the synthesis and interpretation of the review. Studies with greater methodological limitations were interpreted more cautiously, and these limitations were considered when drawing the overall conclusions and identifying evidence gaps.

### 2.7. Data Analysis

A narrative synthesis was conducted because no outcome included a sufficient number of studies with comparable populations, APN interventions, comparators, and outcome measures to support meaningful quantitative pooling. Considerable heterogeneity in study designs, healthcare settings, APN roles, interventions, and outcomes further precluded meta-analysis. Therefore, findings were synthesized narratively according to the review objectives rather than by statistical pooling. Extracted data were organized and summarized according to the review objectives, with particular focus on: (1) the roles and activities of advanced practice nurses, (2) clinical outcomes, (3) patient-reported outcomes, and (4) healthcare service outcomes. Patterns, consistencies, and differences across studies were identified and compared. Findings from different study designs were integrated by synthesizing evidence within these thematic categories and considering the consistency of findings across studies. Where findings differed across studies, these differences were interpreted by considering variations in study design, healthcare settings, APN interventions, and outcome measures.

## 3. Results

### 3.1. Study Characteristics

**Figure 1:**
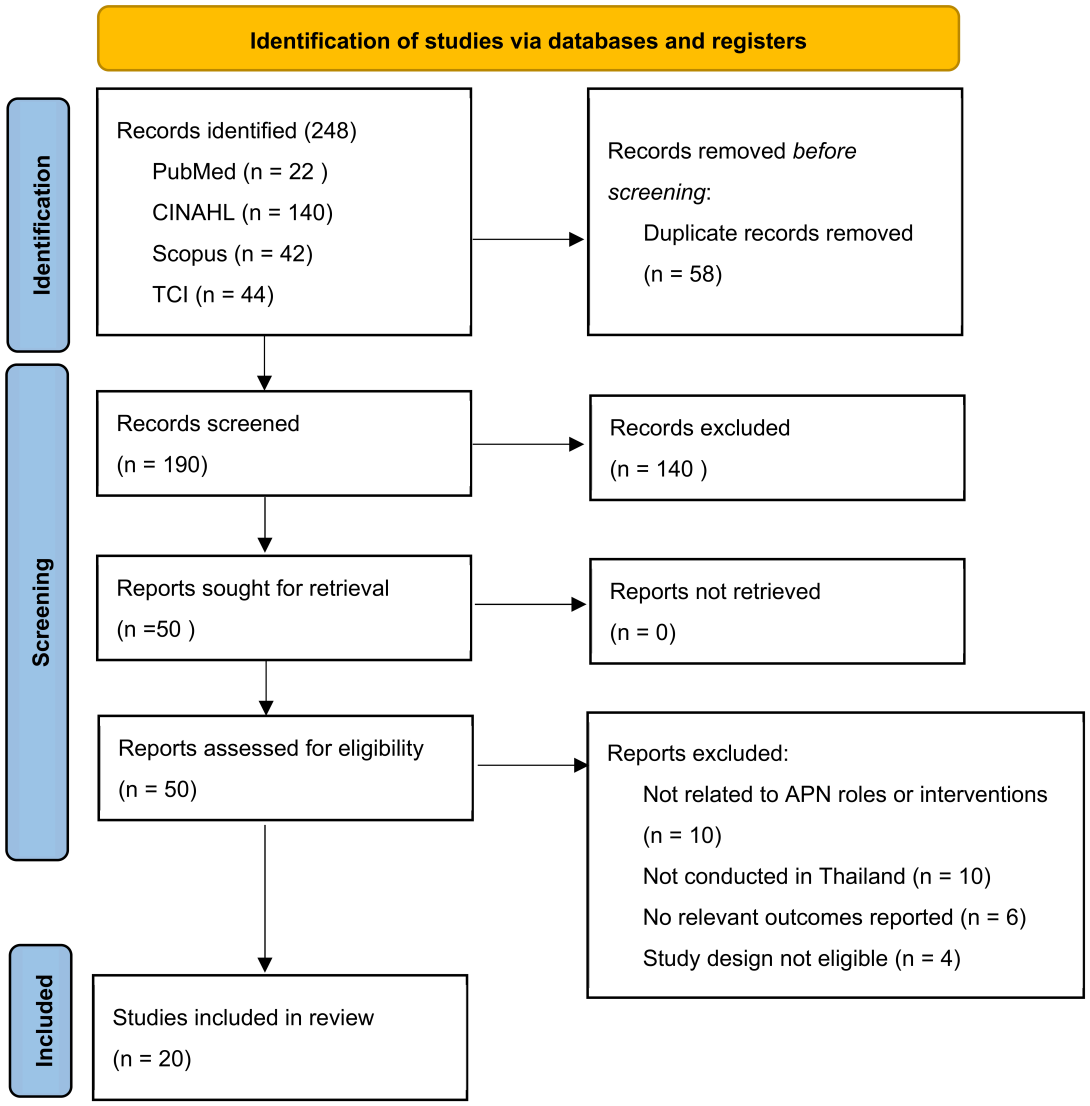
PRISMA 2020 flow diagram of study selection process for the systematic review of advanced practice nursing roles and healthcare outcomes in Thailand.

A comprehensive literature search across four databases yielded 248 records. All identified records were exported and screened for eligibility. After removing 58 duplicate records using EndNote version 21 (Clarivate, London, UK), 190 records remained for title and abstract screening. Of these, 140 records were excluded, and 50 full-text articles were assessed for eligibility. Following full-text review, 30 articles were excluded for the following reasons: not related to APN roles or interventions (*n* = 10), not conducted in Thailand (*n* = 10), no relevant outcomes reported (*n* = 6), and ineligible study design (*n* = 4). A total of 20 studies met the inclusion criteria and were included in the review (Figure 1; Table 1).

A total studies were included in this systematic review, representing a diverse range of study designs, healthcare settings, and advanced practice nursing (APN) specialties in Thailand. The included studies involved diverse units of analysis, including 2388 TNMC-certified APNs, patients, family caregivers, healthcare professionals, stakeholders, and medical records, reflecting the broad scope of APN practice and evaluation in Thailand. The included studies comprised eight quasi-experimental studies, six cross-sectional studies, four mixed-methods studies, one retrospective study, and one qualitative descriptive study, indicating a predominance of non-randomized and observational research designs.

The studies were conducted across a wide range of healthcare settings in Thailand, including tertiary and university hospitals, intensive care units, medical and specialty wards, oncology and palliative care services, psychiatric services, outpatient clinics, primary care centers, community settings, and nationwide Ministry of Public Health contexts [18,19,20,21,22,23,24,25]. The studies represented multiple APN specialty areas. Studies represented multiple APN specialties, including chronic disease management, oncology, critical care, mental health, palliative care, and community health [18,23,24,26,27,28,29,30].

Critical care APN practice was represented by studies in intensive care and mechanically ventilated patients [19,31]. Psychiatric and mental health nursing included studies of schizophrenia care and APN case management [32,33]. Palliative and family-centered care was represented by studies of caregiver training and terminal care management [30,34]. Community and primary care APN roles were reflected in studies of advanced community nurse practitioners and national APN service outputs [20,24]. At the system and workforce levels, several studies examined APN role performance, role implementation, practice situations, career pathway development, and evidence-based practice capacity among APNs across Thailand [21,22,23,24,25,35,36].

**Table 1:** Characteristics of Included Studies on the Roles and Healthcare Outcomes of Advanced Practice Nurses in Thailand (n = 20).

Author/Year	Study Design	Type of APN	Population/Sample	Intervention/Role of APN	Duration/Follow-Up	Outcomes Measured	Key Findings
Wongkpratoom et al. (2010) [35]	Mixed-methods	Multiple specialties	154 APNs in Thailand (Phase 1); 13 APNs interviewed (Phase 2)	APN role development and role performance	Not applicable	Role performance, role development process, factors influencing APN practice.	APNs performed multiple roles including clinical care, education, consultation, administration, and research; barriers included unclear role structure and limited organizational support.
2. Partiprajak et al. (2011) [26]	Mixed-methods	Endocrine/Medical–Surgical	T2DM patients (n = 100; intervention = 44, comparison = 56)	APN-led diabetes support program	Not applicable	BMI, systolic BP, lipid profile, HbA1c, hospitalization, self-care ability, quality of life, and patient satisfaction.	Support-group participants had significantly lower systolic BP and higher self-care ability, quality of life, and satisfaction than the comparison group, with favorable trends in metabolic outcomes.
3. Plaiyod et al. (2012) [27]	Quasi-experimental	Medical–Surgical/Cardiovascular	53 adults with uncontrolled hypertension	APN-led self-care participation program	6 months	Body weight, self-care capability, systolic BP, diastolic BP, BP control.	The APN-led program significantly improved self-care capability and reduced systolic and diastolic blood pressure, with 67.9% achieving blood pressure control.
4. Sonpaveerawong et al. (2012) [32]	Cross-sectional	Psychiatric and Mental Health	1 APN, 51 records, 69 patients/caregivers, 14 stakeholders	APN psychiatric case management	Not applicable	Self-care ability, caregiver burden, satisfaction, readmission, LOS, treatment cost, organizational outcomes.	APN care improved patient self-care and satisfaction, reduced readmission, shortened hospital stay (84.7 vs. 31.6 days), lowered treatment costs (57,826 vs. 21,545 THB), and enhanced service quality and multidisciplinary coordination, while caregivers reported moderate burden.
5. Piboonarluk et al. (2018) [33]	Quasi-experimental	Psychiatric and Mental Health	67 persons with schizophrenia (35 intervention, 32 control)	APN-led case management, psychoeducation, and care coordination	4 weeks	Relapse, psychotic symptoms (BPRS), medication adherence, family expressed emotion.	APN-led case management prevented relapse in persons with schizophrenia, with no relapse in the intervention group compared with four relapse cases in the control group at 4 weeks after discharge.
6. Hanprasitkam et al. (2012) [18]	Mixed-methods	Oncology	1 APN, 139 medical records, 60 breast cancer patients, and 6 stakeholders	APN-led breast cancer care management	Across chemotherapy care trajectory	LOS, complications, readmission, chemotherapy continuity, self-care ability, quality of life, satisfaction, cost, and stakeholder perceptions.	APN-led breast cancer care reduced postoperative readmission to 0%, decreased chemotherapy-related readmission to 0%, improved regular chemotherapy attendance to 84.4%, lowered chemotherapy complications to 33.3%, and achieved high self-care, quality of life, and satisfaction.
7. Thongchai et al. (2012) [19]	Retrospective	Critical Care Medical–Surgical	1 APN and 28 stakeholders (8 nurses, 3 nursing administrators, 1 physician, 6 ICU patients, 10 family members)	APN-led critical care management	Retrospective service outcomes from 2002–2009.	Ventilator days, ventilator weaning time, ICU LOS, complications (VAP, pressure ulcers), ICU readmission, and mortality.	APN-led critical care was associated with reduced ventilator days (6.73 to 4.00), shorter weaning time (95.77 to 2.61 h), lower ICU length of stay (6.18 to 4.30 days), decreased ventilator-associated pneumonia (27.72 to 4.20), and fewer pressure ulcers (6.32 to 0.20), with improved service quality and staff development.
8. Piaseu et al. (2013) [20]	Mixed-methods	Community Health	405 service users (200 ACNP, 205 GNP) + 13 professionals/stakeholders	Community-based APN care	Cross-sectional service evaluation during the study period.	Healthcare service outcomes (health status, healthcare expenses). and patient satisfaction (overall satisfaction, accessibility, professional competence).	ACNP provided broader services and higher satisfaction compared with GNP.
9. Tungpunkom et al. (2016) [21]	Cross-sectional	Multiple specialties	566 APNs	APN role enactment survey	Not applicable	Supporting factors and barriers to APN role performance.	Organizational policy support and supervision facilitated APN roles, while unclear roles and limited resources were barriers.
10. Thamasarangkul et al. (2016) [22]	Cross-sectional	Internal Medicine Nursing	2 nursing administrators, 4 health personnel, 8 ward/unit head nurses, 15 APNs, and 2 patients/families	APN role evaluation	Not applicable	Structure, process, and outcomes of APN practice.	APN roles contributed to improved service quality and healthcare delivery.
11. Jerawatana et al. (2018) [28]	Quasi-experimental	Medical–Surgical/Endocrine	Patients with T2DM with complex problems (n = 30)	APN-led diabetes intervention	10 weeks	Diabetes knowledge, self-care behavior, quality of life, HbA1c.	Knowledge, self-care behavior, and quality of life improved and HbA1c decreased significantly.
12. Singprasert et al. (2018) [29]	Quasi-experimental	Nephrology	Patients with pre-dialysis CKD (n = 75)	APN-led behavior modification program	6 months	CKD knowledge, self-care behavior, kidney function indicators.	Knowledge and self-care improved and kidney function indicators showed improvement.
13. Semsarn et al. (2018) [34]	Quasi-experimental	Adult-Gerontological, Palliative Care	Caregivers of patients with terminal illness (n = 30)	APN-led caregiver training	Not applicable	Caregiver confidence, caregiver stress, caregiver satisfaction, patient outcomes (advance care planning, ED visits).	Confidence and satisfaction increased while caregiver stress decreased significantly.
14. Rhiantong et al. (2019) [37]	Quasi-experimental	Adult-Gerontological/Cardiovascular	Patients with heart failure (n = 71)	APN-led continuing care	3 months	Functional status, quality of life, hospital LOS, healthcare costs.	Functional status and quality of life improved while LOS and costs decreased.
15. Akkadechanunt et al. (2021) [23]	Qualitative descriptive	Multiple APN specialties (community, adult-gerontology, psychiatric, anesthesia, maternal–child, infection control)	Certified APNs across Thailand (n = 27)	APN role performance	Not applicable	Role performance and factors influencing APN practice.	APNs reported performing nine key roles including direct patient care, multidisciplinary collaboration, education and mentoring, consultation, quality improvement, patient advocacy, evidence-based practice leadership, outcome evaluation, and healthcare system development.
16. Rutwongsa et al. (2021) [24]	Cross-sectional	Multiple specialties	711 APNs under Ministry of Public Health	National APN workforce survey	Not applicable	Practice characteristics, distribution by specialty and setting, numbers of work outputs, dissemination/publication of APN work.	Most APNs were female, aged 41–50 years and worked in medical-surgical nursing. A total of 1031 work outputs were identified, with the highest proportion in medical-surgical nursing and primary care/district health systems. Most dissemination occurred at the national level, but many outputs had not been formally published.
17. Temthup et al. (2021) [30]	Quasi-experimental	Palliative Care	Patients with terminal hepatocellular carcinoma (n = 36)	APN-led nurse case management	5 days	Symptom-induced suffering, palliative care outcomes.	Symptom-related suffering decreased significantly and palliative outcomes improved.
18. Rakhab et al. (2021) [25]	Cross-sectional	Multiple specialties	333 APNs	APN role and service evaluation	Not applicable	APN role performance, clinical activities, service outputs.	APNs provided direct clinical care, consultation, education, and system improvement activities, contributing to improved service efficiency and quality of care in healthcare settings.
19. Bhatarasakoon et al. (2022) [36]	Cross-sectional	Multiple specialties	566 APNs across 10 specialties in Thailand	APN evidence-based practice survey	Not applicable	EBP knowledge/skills, attitudes toward EBP, EBP practice.	APNs demonstrated positive attitudes toward evidence-based practice but reported lower levels of EBP knowledge and research skills; higher education and work experience were associated with greater EBP use.
20. Supreeyatitikul et al. (2025) [31]	Quasi-experimental	Critical Care	49 patients receiving mechanical ventilation in the ICU (24 intervention, 25 comparison)	APN-led respiratory muscle weakness prevention program	2-week intervention with outcomes measured during the program and at hospital discharge	MIP, ventilation duration, weaning duration, weaning success, ICU LOS, hospital LOS, and healthcare costs.	The APN-led Respiratory Muscle Weakness Prevention Program improved MIP and weaning success (100% vs. 72%), reduced ventilation duration (5.14 vs. 17.48 days), shortened weaning duration (2.15 vs. 6.54 days), decreased ICU LOS (6.96 vs. 12.67 days), reduced hospital LOS (17.27 vs. 34.04 days), and lowered healthcare costs (USD 1335.84 vs. 3147.36).

APN = advanced practice nurse; LOS = length of stay; ICU = intensive care unit; CKD = chronic kidney disease; T2DM = type 2 diabetes mellitus; BP = blood pressure; BMI = body mass index; MIP = inspiratory muscle strength.

### 3.2. Roles of Advanced Practice Nurses in Thailand

Across studies, APNs in Thailand functioned beyond direct bedside care and demonstrated multidimensional roles at patient, team, and system levels. National role development studies reported strong APN performance in direct care, educator, consultant, administrator, and researcher domains, with somewhat lower performance in ethicist/legalist roles [35]. A nationwide qualitative study further identified nine core APN roles: direct care, multidisciplinary collaboration, patient education and mentoring, consultation, quality improvement, advocacy and ethical decision-making, implementation of evidence-based practice, outcome evaluation, and service system development [23]. In primary care settings, APNs demonstrated broader scope of practice than general nurse practitioners, including comprehensive assessment, prescribing, referral, home visits, preventive programs, and community leadership initiatives [20].

### 3.3. Clinical Outcomes in Chronic Disease Management

Several studies demonstrated favorable outcomes associated with APN-led chronic disease management.

In people with type 2 diabetes, an APN-led support group model was associated with improved blood pressure control, self-care ability, quality of life, and patient satisfaction [26]. Among patients with uncontrolled hypertension, a six-month APN-led self-care participation program was associated with significantly improved self-care capability and reduced systolic and diastolic blood pressure, with 67.9% achieving blood pressure control [27]. In patients with complex diabetes problems, a 10-week APN-led intervention was associated with reduced HbA1c levels, improved diabetes knowledge, enhanced self-care behaviors, and increased quality of life [28]. Among patients with chronic kidney disease, a six-month APN-led behavioral modification program was associated with improvements in disease knowledge, self-care behavior, readiness for change, and selected clinical indicators [29]. For patients with heart failure, APN-led care was associated with improvements in functional status, quality of life, and satisfaction while reducing hospital length of stay and healthcare costs [37].

#### 3.3.1. Mental Health Outcomes

Two studies in psychiatric settings reported consistent benefits of APN involvement in schizophrenia care. APN-integrated services were associated with lower readmission rates, shorter hospital stays, lower treatment costs, higher self-care ability, and greater patient satisfaction [32]. In addition, APN-led case management significantly reduced early relapse after discharge compared with usual care [33].

#### 3.3.2. Oncology and Palliative Care Outcomes

In women with breast cancer, APN-led care was associated with fewer chemotherapy-related complications, improved treatment continuity, lower readmission rates, enhanced self-care ability, higher quality of life, and greater satisfaction with care [18]. Among patients with terminal hepatocellular carcinoma, an APN-led nurse case management program was associated with reduced symptom-induced suffering and improved palliative care outcomes [30]. Among terminally ill patients and their caregivers, an APN-led caregiver training program improved caregiver confidence, reduced stress, increased satisfaction, facilitated advance care planning, and supported peaceful home death outcomes [34].

#### 3.3.3. Critical Care Outcomes

Evidence from critical care settings also demonstrated a strong APN impact. A case study in an intensive care unit reported reductions in ICU length of stay, ventilator days, ventilator-associated pneumonia, pressure ulcers, and improvements in staff competency and evidence-based care processes following APN service implementation [19]. More recent evidence showed that an APN-led respiratory muscle weakness prevention program was associated with improvements in inspiratory muscle strength, shortened ventilator duration, increased weaning success, reduced ICU and hospital length of stay, and lowered healthcare costs among mechanically ventilated patients [31].

### 3.4. Workforce Development, Career Pathways, and System Barriers

System-level studies consistently indicated that APN effectiveness depended on organizational support and clear role structures. A national survey found that many APNs were not deployed in full advanced practice roles and faced role ambiguity, heavy workload, and lack of formal positions. Administrative support, leadership, and institutional recognition were key facilitators [21]. A tertiary hospital evaluation similarly reported that APNs practiced across all national competencies but often combined APN responsibilities with routine nursing duties or used personal time to complete advanced practice work [22]. Another national survey showed that unclear career pathways, limited mentorship, insufficient funding, and weak research infrastructure constrained long-term APN professional development [25]. At the national service level, 711 APNs under the Ministry of Public Health reported 1031 professional outputs, particularly in primary care, district health systems, non-communicable disease care, and mental health services [24].

### 3.5. Evidence-Based Practice Capacity

A multi-setting survey of 566 APNs found highly positive attitudes toward evidence-based practice and moderate implementation in routine care. However, lower scores were reported for research appraisal, information technology skills, evidence retrieval, and judging the validity of research findings. Higher education and greater experience were associated with stronger evidence-based practice competency [36].

### 3.6. Overall Synthesis of Findings

Overall, the included studies addressed two principal areas: the roles performed by APNs and the healthcare outcomes associated with APN practice. APNs performed multidimensional clinical, educational, consultative, coordinating, leadership, and evidence-based practice roles across diverse healthcare settings. The implementation and sustainability of these roles were influenced by organizational support, role clarity, career structures, protected time, and access to research and evidence-based practice resources. Although APN-led models were generally associated with favorable patient-, caregiver-, and service-level outcomes, the predominance of non-randomized and observational study designs limits causal interpretation.

### 3.7. Quality Appraisal

The methodological quality of the included studies was assessed using the Mixed Methods Appraisal Tool (MMAT, 2018) for mixed-methods studies [17] and the Joanna Briggs Institute (JBI) critical appraisal checklists for quasi-experimental, analytical cross-sectional, and qualitative studies [14,15,16]. Overall, the included studies demonstrated moderate to high methodological quality and were considered suitable for inclusion in the synthesis.

*Mixed-Methods Studies*

The methodological quality of the five mixed-methods studies was generally high (Supplementary File Table S1). All studies clearly stated their research questions, collected data appropriate to address these questions, provided an adequate rationale for using a mixed-methods design, effectively integrated qualitative and quantitative components, appropriately interpreted integrated findings, and met the quality criteria of the respective methodological traditions. However, three studies did not clearly address divergences or inconsistencies between quantitative and qualitative findings, representing the primary methodological limitation within this group.

*Quasi-Experimental Studies*

Overall, the quasi-experimental studies demonstrated good methodological quality (Supplementary File Table S2). All studies clearly identified the cause-and-effect relationship, conducted multiple outcome measurements before and after the intervention, measured outcomes consistently and reliably, reported adequate follow-up, and used appropriate statistical analyses. Five studies included a control group, whereas four employed a single-group design, rendering group comparisons inapplicable. Among studies with comparison groups, participants were generally comparable and received similar care, except for the intervention of interest. The primary methodological limitation was the absence of a control group in several studies.

*Cross-Sectional Studies*

The methodological quality of the cross-sectional studies was generally satisfactory (Supplementary File Table S3). All studies clearly defined inclusion criteria, used valid and reliable measures of exposure and outcomes, applied appropriate statistical analyses, and adequately described study participants and settings. However, three studies did not clearly identify potential confounding factors or report strategies to address them. Consequently, inadequate consideration of confounding represented the main methodological concern among the cross-sectional studies.

*Qualitative Study*

The included qualitative study demonstrated good methodological quality across most appraisal domains (Supplementary File Table S4). The study demonstrated congruity among the research methodology, research questions, data collection methods, data analysis, and the interpretation of findings. Participants’ voices were adequately represented, ethical approval was obtained, and conclusions were supported by the data. However, the philosophical perspective underpinning the study was not clearly described, and the researcher’s cultural or theoretical positioning and influence on the research process were not addressed. These issues represented the main methodological limitations of the qualitative study.

Overall, the primary methodological concerns across the included studies were related to the management of confounding factors, the absence of control groups in some quasi-experimental studies, limited discussion of inconsistencies between qualitative and quantitative findings in mixed-methods studies, and insufficient reporting of researcher reflexivity in the qualitative study.

## 4. Discussion

This systematic review provides a country-specific synthesis of the roles performed by APNs and the healthcare outcomes associated with APN practice in Thailand. Two overarching findings emerged. First, APN practice extended beyond direct clinical care to include education, consultation, care coordination, leadership, and evidence-based practice. Second, APN-led models were generally associated with favorable patient-, caregiver-, and service-level outcomes. These findings are broadly consistent with international evidence on APN practice [9,38]. However, direct comparison should be interpreted cautiously because APN regulation, educational preparation, prescribing authority, professional autonomy, and scope of practice differ across healthcare systems.

### 4.1. Roles of APNs in the Thai Healthcare System

The findings indicate that APN practice in Thailand is multidimensional and extends beyond traditional bedside nursing. This scope is consistent with contemporary international APN frameworks, which conceptualize advanced practice nursing as the integration of expert clinical care, leadership, education, consultation, research, and system-level competencies [5,39].

The adaptability of APN roles across hospitals, intensive care units, primary care, oncology, psychiatric care, and palliative services may be particularly relevant to Thailand as the health system responds to population ageing, multimorbidity, and increasing demand for coordinated long-term care. International literature similarly identifies APNs as important providers for patients with complex healthcare needs across hospital and community settings [40]. However, the extent to which APNs can perform these roles is likely to depend on regulatory clarity, organizational recognition, protected time, and opportunities to practice within their formally recognized scope.

### 4.2. Clinical and Healthcare Outcomes

The most consistent outcome evidence was identified in chronic disease and acute care settings. These findings are broadly consistent with international systematic reviews reporting favorable patient- and service-level outcomes associated with APN practice [6,9].

One possible explanation is that APN-led models commonly incorporate comprehensive assessment, individualized care planning, patient education, continuity of follow-up, and interdisciplinary coordination. These mechanisms may support treatment adherence, timely recognition of clinical deterioration, and continuity of care. Nevertheless, the Thai evidence was derived largely from single-center, quasi-experimental, or observational studies, and several interventions were implemented within multidisciplinary care teams. The independent contribution of APNs therefore cannot be determined with certainty [26,27,28,29,37]. Furthermore, differences in healthcare systems, APN regulation, and professional autonomy should be considered when comparing these findings with international evidence.

### 4.3. Family-Centered and Palliative Care Outcomes

The inclusion of family and caregiver outcomes highlights an important contextual feature of APN practice in Thailand, where family members often have central responsibilities in long-term and end-of-life care. APN involvement may be particularly relevant because education, symptom guidance, emotional support, and care coordination can extend beyond the individual patient to the broader family caregiving system.

This interpretation is consistent with wider evidence indicating that caregiver-focused nursing interventions may reduce psychological distress and caregiving burden while improving quality of life [41]. However, the Thai evidence was derived from relatively small studies with limited comparative designs; therefore, these findings should be interpreted as preliminary evidence of potential benefit rather than established causal effects.

### 4.4. Workforce, Career Pathways, and System Barriers

The findings suggest that APN implementation in Thailand is strongly shaped by organizational and regulatory context. Role ambiguity, limited protected time, unclear career progression, and inconsistent institutional recognition may restrict the extent to which APNs can perform advanced roles, regardless of their formal preparation or certification [21,22,25,35]. These contextual factors may also explain variation in APN role implementation across healthcare settings.

International evidence similarly indicates that regulatory clarity, leadership engagement, clearly defined scopes of practice, and organizational integration are important conditions for successful APN implementation [42]. Compared with healthcare systems in which APNs have greater professional autonomy, implementation in Thailand remains more dependent on local institutional policies and leadership support.

The findings therefore support further evaluation of APN role implementation and suggest that clearer career structures, formal APN positions, standardized scopes of practice, and supportive leadership may facilitate APN practice in Thailand. These implications should be considered context-dependent and should not be interpreted as evidence that workforce investment alone will necessarily improve national health system performance or reduce costs. The World Health Organization has also identified advanced practice nursing as a potentially important component of future health workforce development [3].

### 4.5. Evidence-Based Practice and Research Capacity

This review also indicates that EBP capability remains an important area for APN workforce development, particularly in research appraisal, evidence retrieval, and digital information skills. Because evidence-based practice is a core component of advanced nursing practice, limited competence in these areas may restrict the ability of APNs to evaluate and translate research findings into routine care.

Previous evidence suggests that APN evidence-based healthcare competence is influenced by education, organizational support, mentorship, and access to evidence resources [43,44]. Therefore, strategies to strengthen EBP should address not only individual knowledge and skills but also access to evidence, protected time, mentoring, and supportive organizational cultures. Similar needs have been identified among APNs in Thailand [24].

Future research should prioritize multicenter studies, larger samples, rigorous comparative designs, and standardized outcome measures. Such studies are needed to clarify the independent contribution of APN practice and provide a stronger basis for future workforce and policy decisions.

### 4.6. Strengths and Limitations

This review has several strengths. It synthesized evidence across diverse specialties and care settings and captured patient-, family-, provider-, and system-level outcomes. It also focused specifically on Thailand, providing contextually relevant evidence for national planning. Importantly, many of the limitations identified in this review originated from the primary studies, including their study designs, sample sizes, and methodological variability, which directly affected the certainty and interpretation of the overall evidence.

However, several limitations should be acknowledged. Many included studies used quasi-experimental or descriptive designs, and only a limited number employed comparative designs, thereby restricting the overall strength of the available evidence and limiting causal inference. Sample sizes were often modest, and methodological heterogeneity precluded meta-analysis. The substantial variation in study designs, APN interventions, healthcare settings, and outcome measures reduced the certainty of the evidence and may limit the external validity and transferability of the findings across different healthcare settings. In addition, several included studies evaluated APN-led interventions delivered within multidisciplinary care teams. Therefore, the observed improvements may reflect the combined contributions of APNs and other healthcare professionals, making it difficult to isolate the independent effect of APN practice. Some studies were published in local journals with variable reporting detail. Therefore, although the overall direction of findings was consistently positive, effect sizes should be interpreted cautiously. Furthermore, the exclusion of grey literature, the inclusion of only English- and Thai-language publications, and the possibility of publication bias and selective reporting may have influenced the completeness of the available evidence and may partly explain the predominance of favorable findings across the included studies. Although methodological quality was considered during the synthesis, the heterogeneity of the study designs and appraisal tools precluded formal stratification of the conclusions according to methodological quality. Accordingly, the findings of this review should be interpreted as demonstrating consistent associations rather than definitive evidence of causal effects, and the overall strength of the evidence should be considered moderate because it is based predominantly on observational and quasi-experimental studies. Therefore, although the available evidence suggests generally favorable associations with APN practice, uncertainty remains regarding the magnitude, consistency, and independent contribution of APNs across different settings and populations.

## 5. Conclusions

APNs in Thailand appear to contribute positively to healthcare quality, patient outcomes, family support, and service efficiency across multiple settings. However, these findings should be interpreted with caution because the available evidence is derived primarily from quasi-experimental and observational studies. Consequently, the reported findings should be interpreted as evidence of consistent associations rather than definitive causal effects. Realizing the full value of APNs will require stronger career structures, clearer policy support, and continued investment in education and evidence-based practice capacity. Strengthening the APN workforce may be considered as one potential strategy; however, further high-quality evidence is needed before firm policy recommendations can be made regarding its impact on national health system performance.

## Data Availability

No new data were created or analyzed in this study. Data sharing is not applicable to this article.

## Supplementary Materials

The following supporting information can be downloaded at https://www.mdpi.com/article/10.3390/healthcare14152300/s1, Table S1: MMAT. Table S2: Quasi-experimental Studies. Table S3: Cross-sectional. Table S4: Qualitative. Table S5: Search Strategy (Example).
