# Supplementary material for: Roles and Outcomes of Advanced Practice Nurses in Thailand: A Systematic Review
Source: Healthcare (Basel). 2026 Jul 29;14(15):2300. doi: 10.3390/healthcare14152300 (PMC13465538; doi:10.3390/healthcare14152300)
Supplement: Supplementary file 1 [file healthcare-14-02300-s001.zip › healthcare-4412842-supplementary.pdf]

Critical Appraisal

Table S1: MMAT

**Study**	**Q1**	**Q2**	**Q3**	**Q4**	**Q5**	**Q6**	**Q7**
Wongkpratoom et al. (2010)	Y	Y	Y	Y	Y	C	Y
Partiprajak et al. (2011)	Y	Y	Y	Y	Y	C	Y
Hanprasitkam et al. (2012)	Y	Y	Y	Y	Y	Y	Y
Thongchai et al. (2012)	Y	Y	Y	Y	Y	C	Y
Piaseu et al. (2013)	Y	Y	Y	Y	Y	Y	Y

Note. Y = Yes; N = No; C = Can't tell. Studies were appraised using the Mixed Methods Appraisal Tool (MMAT), version 2018.

Q1. Are there clear research questions?

Q2. Do the collected data allow to address the research questions?

Q3 Is there an adequate rationale for using a mixed methods design to address the research question?

Q4. Are the different components of the study effectively integrated to answer the research question?

Q5. Are the outputs of the integration of qualitative and quantitative components adequately interpreted?

Q6. Are divergences and inconsistencies between quantitative and qualitative results adequately addressed?

Q7. Do the different components of the study adhere to the quality criteria of each tradition of the methods involved?

Table S2: Quasi-experimental Studies

Study	Q1	Q2	Q3	Q4	Q5	Q6	Q7	Q8	Q9
Plaiyod et al. (2012)	Y	Y	NA	NA	Y	Y	Y	Y	Y
Sonpaveerawong et al. (2012)	Y	Y	Y	Y	Y	Y	Y	Y	Y
Piboonarluk et al. (2018)	Y	Y	Y	Y	Y	Y	Y	Y	Y
Jerawatana et al. (2018)	Y	N	NA	NA	Y	Y	Y	Y	Y
Singprasert et al. (2018)	Y	N	NA	NA	Y	Y	Y	Y	Y
Semsarn et al. (2018)	Y	N	NA	NA	Y	Y	Y	Y	Y
Rhiantong et al. (2019)	Y	Y	Y	Y	Y	Y	Y	Y	Y
Temthup et al. (2021)	Y	N	NA	NA	Y	Y	Y	Y	Y
Supreeyatitikul et al. (2025)	Y	Y	Y	Y	Y	Y	Y	Y	Y

1 Is it clear in the study what is the "cause" and what is the "effect" (i.e. there is no confusion about which variable comes first)?

2 Was there a control group?

3 Were participants included in any comparisons similar?

4 Were the participants included in any comparisons receiving similar treatment/care, other than the exposure or intervention of interest?

5 Were there multiple measurements of the outcome, both pre and post the intervention/exposure?

6 Were the outcomes of participants included in any comparisons measured in the same way?

7 Were outcomes measured in a reliable way?

8 Was follow-up complete and if not, were differences between groups in terms of their follow-up adequately described and analyzed?

9 Was appropriate statistical analysis used?

Table S3: Cross-sectional

Study	Q1	Q2	Q3	Q4	Q5	Q6	Q7	Q8
Tungpunkom et al. (2016)	Y	Y	Y	Y	U	N	Y	Y
Thamasarangkul et al. (2016)	Y	Y	Y	Y	U	N	Y	Y
Rutwongsa et al. (2021)	Y	Y	Y	Y	U	N	Y	Y
Rakhab et al. (2021)	Y	Y	Y	Y	Y	Y	Y	Y
Bhatarasakoon et al. (2022)	Y	Y	Y	Y	Y	Y	Y	Y

Note. Y = Yes; N = No; U = Unclear; NA = Not applicable.

1 Were the criteria for inclusion in the sample clearly defined?

2 Were objective, standard criteria used for measurement of the condition?

3 Was the exposure measured in a valid and reliable way?

4 Were the outcomes measured in a valid and reliable way?

5 Were confounding factors identified?

6 Were strategies to deal with confounding factors stated?

7 Was appropriate statistical analysis used?

8 Were the study subjects and the setting described in detail?

Table S4: Qualitative

Study	Q1	Q2	Q3	Q4	Q5	Q6	Q7	Q8	Q9	Q10
Akkadechanunt et al. (2021)	U	Y	Y	Y	Y	N	N	Y	Y	Y

Q1 Is there congruity between the stated philosophical perspective and the research methodology?

Q2 Is there congruity between the research methodology and the research question or objectives?

Q3 Is there congruity between the research methodology and the methods used to collect data?

Q4 Is there congruity between the research methodology and the representation and analysis of data?

Q5 Is there congruity between the research methodology and the interpretation of results?

Q6 Is there a statement locating the researcher culturally or theoretically?

Q7 Is the influence of the researcher on the research, and vice-versa, addressed?

Q8 Are participants, and their voices, adequately represented?

Q9 Is the research ethical according to current criteria or approved by an appropriate body?

Q10 Do the conclusions flow from the analysis or interpretation of the data?

Table S5. Search Strategy (Example).

Search conducted on… PubMed**…** Date…31 January 2026.

Diabetes mellitus

PICO		Search Terms	Number of Studies
Population	1.	advanced practice nurse	1380
	2.	advanced practice nursing	431
	3.	clinical nurse specialist	2088
	4.	nurse practitioner	8205
	5.	advanced practice	9373
	6.	clinical nurse specialist	2088
	7.	APN	3416
	8.	CNS	140,866
	9.	1OR 2 OR3 OR4 OR5 OR6 OR7 OR8	161,225
outcome	10.	clinical outcome	101,790
	11.	health outcome	6221
	12.	patient outcome	20,484
	13.	effectiveness	786,640
	14.	quality of life	477,637
	15.	satisfaction	231,495
	16.	healthcare utilization	10,417
	17.	length of stay	98,429
	18.	10 OR11 OR13 OR14 OR15 OR16 OR 17	3,199,747
	19.	Thailand	40,562
	20.	Thai	21,231
	21.	20 OR 21	52,542
	22.	9 AND 19 AND 22	16
